# Tensor-Based ECG Anomaly Detection toward Cardiac Monitoring in the Internet of Health Things

**DOI:** 10.3390/s21124173

**Published:** 2021-06-17

**Authors:** Houliang Zhou, Chen Kan

**Affiliations:** Department of Industrial, Manufacturing, and Systems Engineering, The University of Texas at Arlington, Arlington, TX 76019, USA; houliang.zhou@mavs.uta.edu

**Keywords:** cardiac monitoring, Internet of Health Things, tensor decomposition, anomaly detection, statistical process monitoring

## Abstract

Advanced heart monitors, especially those enabled by the Internet of Health Things (IoHT), provide a great opportunity for continuous collection of the electrocardiogram (ECG), which contains rich information about underlying cardiac conditions. Realizing the full potential of IoHT-enabled cardiac monitoring hinges, to a great extent, on the detection of disease-induced anomalies from collected ECGs. However, challenges exist in the current literature for IoHT-based cardiac monitoring: (1) Most existing methods are based on supervised learning, which requires both normal and abnormal samples for training. This is impractical as it is generally unknown when and what kind of anomalies will occur during cardiac monitoring. (2) Furthermore, it is difficult to leverage advanced machine learning approaches for information processing of 1D ECG signals, as most of them are designed for 2D images and higher-dimensional data. To address these challenges, a new sensor-based unsupervised framework is developed for IoHT-based cardiac monitoring. First, a high-dimensional tensor is generated from the multi-channel ECG signals through the Gramian Angular Difference Field (GADF). Then, multi-linear principal component analysis (MPCA) is employed to unfold the ECG tensor and delineate the disease-altered patterns. Obtained principal components are used as features for anomaly detection using machine learning models (e.g., deep support vector data description (deep SVDD)) as well as statistical control charts (e.g., Hotelling $T^{2}$ chart). The developed framework is evaluated and validated using real-world ECG datasets. Comparing to the state-of-the-art approaches, the developed framework with deep SVDD achieves superior performances in detecting abnormal ECG patterns induced by various types of cardiac disease, e.g., an F-score of 0.9771 is achieved for detecting atrial fibrillation, 0.9986 for detecting right bundle branch block, and 0.9550 for detecting ST-depression. Additionally, the developed framework with the *T*^2^ control chart facilitates personalized cycle-to-cycle monitoring with timely detected abnormal ECG patterns. The developed framework has a great potential to be implemented in IoHT-enabled cardiac monitoring and smart management of cardiac health.

## 1. Introduction

The contraction of heart chambers is coordinated by the cardiac electrical activity. Under normal condition, an electrical stimulus is generated by the pacemaker, i.e., the sinus node, to activate both atria. The stimulus then travels down to the atrioventricular node and propagates through the bundle of His–Purkinje fibers to stimulate the contraction of left and right ventricles [1]. With the onset of cardiac disease, the function of the sinus node and/or the conduction pathway will be interrupted, thereby affecting the normal cardiac electrical activity [2]. It is imperative to monitor the cardiac electrical activity and identify the disease-altered patterns for the detection of cardiac disease.

For decades, the electrocardiogram (ECG) has been extensively used as an indispensable procedure for the monitoring of cardiac electrical activity [3]. In clinical settings, multiple electrodes are attached on the body surface to record the cardiac electrical activity from different directions. As such, multi-channel ECG signals, i.e., 3-lead ECG or 12-lead ECG, are recorded, which are considered as the gold standard for the diagnosis of cardiac disease. With advances in sensing and communication technologies, wearable devices with more tolerable sensors (e.g., textiles [4] and skin-like sensors [5]) have been increasingly developed to record the ECG in out-of-hospital settings. In particular, the advent of the Internet of Health Things (IoHT) prompts the integration of a multitude of sensors, computing units, and databases, together with patients and physicians in a networked structure. For cardiac care, the IoHT empowers anywhere-and-anytime cardiac health data collection and processing, which unleashes a great potential for continuous patient-centered monitoring and early detection of cardiac anomalies [6,7].

Realizing the full potential of IoHT-enabled cardiac monitoring hinges, to a great extent, on the information processing of collected ECGs. Notably, the pattern of ECG signals reveals rich information pertinent to the underlying cardiac condition. For example, absent P wave or irregular R-R intervals suggest the onset of atrial fibrillation, and inverted T wave is oftentimes associated with the occurrence of myocardial infarction [1]. In the past, cardiologists were accustomed to manually analyzing the ECG and interpreting disease-altered patterns. The past decade has witnessed the increasing uptake of computer programs for automatic ECG analysis. In the literature, statistical models and machine learning algorithms have been developed for ECG pattern recognition and anomaly detection. For example, Yang et al. quantified the similarity in the ECG morphology for the detection of myocardial infarctions. Based on the similarities, ECG signals were embedded into a graph, and the node coordinates were used as features for classification [1]. Tripathy et al. decomposed ECG signals using Fourier–Bessel series expansion-based empirical wavelet transform. Descriptive statistics such as skewness and entropy were then extracted from the wavelet coefficients for the identification of myocardial infarction [8]. With recent advances in deep learning, deep neural networks have been increasingly leveraged for ECG anomaly detection. Mathews et al. employed the Restricted Boltzman Machine and deep belief network for the identification of arrhythmias in single-lead ECG [9]. Peimankar and Puthusserypady deployed the convolutional neural network (CNN) and long short-term memory (LSTM) network for the segmentation of P, QRS, and T waves from ECG signals [10]. For more reviews on ECG pattern recognition algorithms, please refer to [11].

However, most existing algorithms for ECG pattern recognition (e.g., above-reviewed algorithms) are based on supervised learning. A large number of ECG signals with labels (i.e., the diagnostic information such as normal, abnormal, or a specific type of disease) are required for the training of these algorithms. To obtain the labels, it requires cardiologists to manually assess the ECG, which is labor-intensive and time-consuming. In the IoHT, continuous cardiac monitoring is performed, and ECG signals are recorded for days, weeks, and months [6]. It is prohibitive to manually label the ECG signals to implement the supervised learning methods. More importantly, it is impractical to extensively collect and manually label abnormal ECG cycles so that supervised algorithms can be trained. In most cases, only the normal condition of a specific patient is available when the monitoring starts. It is generally unknown about when and what type of cardiac disorders would be onset. Notably, variations exist among patients and it is important to focus on patient-specific information for the detection of subtle changes in cardiac conditions, especially at the early stage.

Therefore, semi-supervised and unsupervised algorithms are increasingly available for ECG analytics. For example, Zhai et al. proposed a semi-supervised learning approach for the detection of ventricular ectopic beats in the ECG. By examining the correlation in spectrograms, normal cycles were identified in an unsupervised manner. They were then used to train a semi-supervised learning model to distinguish disease-altered ECG cycles [12]. Cardona et al. employed Gaussian mixtures for the modeling of ECG signals, and a K-nearest neighbors clustering approach was used to separate normal from left bundle branch block ECG samples [13]. He et al. processed the ECG signals with entropy-based principal component analysis (PCA). The extracted principal components were fed into fuzzy c-means clustering for unsupervised detection of abnormal ECG patterns [14].

In addition, statistical control charts are oftentimes treated as unsupervised approaches for process monitoring and anomaly detection. The goal of a control chart is to identify nonconformities by comparing the monitoring statistics with control limits that are established using normal samples. Although early studies focused on quality control in manufacturing [15,16,17], statistical control charts have been increasingly extended to monitor various processes, such as cyber-attacks [18] and project management [19]. In particular, statistical control charts are widely used in monitoring healthcare systems and biological processes. For example, Sogandi et al. developed a Bernoulli group exponential weighted moving average control chart to capture state changes of papillary thyroid cancer. Dynamic probability control limits were deployed to improve the algorithm’s applicability [20]. In [21], the authors deployed a Hotelling $T^{2}$ control chart for cycle-to-cycle monitoring of ECG. ECG signals were represented in a 3D state space in which heterogeneous recurrence measures were calculated and used as features for the control chart.

Notably, disease-altered cardiac electrical activity manifests complex patterns, and effective IoHT-based cardiac monitoring calls for the adoption of advanced machine learning and deep learning algorithms. However, the 1D ECG signal poses a significant challenge for adopting a richer body of advanced machine learning algorithms that are designed for 2D images and higher-dimensional data. Even for existing deep learning based ECG analysis, many of them still follow the flow of “feature extraction and classification”. In other words, time and/or frequency domain features are first extracted. Then, the features are fed into deep neural networks for classification. Some studies developed end-to-end learning architectures that directly fed the collected ECG signals to the CNN model by considering the signal amplitudes as features [22]. This is, nevertheless, not only vulnerable to noises, but also hinders the adoption of powerful high-dimensional convolution operations in deep learning. A few recent approaches attempted to address such challenge by representing the ECG using image data. For example, Naz et al. [23] segmented the 1D ECG into equi-sized segments and reshaped each segment into a 2D matrix (i.e., image). Du et al. [24] and Hao et al. [25] analyzed the ECG papers as images using deep neural networks. Some other works transformed 1D ECGs into gray-scale images by considering ECG traces as black pixels, while the background were filled with white pixels [26]. In addition, Ullah et al. [27] used continuous wavelet to decompose the 1D ECG and stacked the obtained wavelet coefficients (along multiple scales) as a 2D image. Notably, it is difficult to fully preserve the diagnostic information in the ECG by using spatial information of pixels in the converted image (e.g., [23,24,25,26]), as the time and frequency information in the ECG signal could be blurred and interrupted. Additionally, the spectrogram representation of ECG (i.e., [27]) generates redundant information in the converted image (due to the continuous change in the position and scale of the wavelet), which makes the spatial correlation of image pixels less interpretable. 

In this study, a new framework is developed to leverage tensor representation of multi-channel ECG for unsupervised anomaly detection. Specifically, a new time series imaging approach is investigated to cast the ECG signal into a high-dimensional tensor. Further, we leverage a low-rank tensor decomposition approach to unfold the ECG tensor and explore the disease-induced abnormalities. The learned tensor-to-vector projection facilitates the extraction of pertinent features about the underlying cardiac dynamics, which can be fed into unsupervised machine learning algorithms and statistical control charts for condition monitoring and anomaly detection. Our major contributions are summarized as follows:

1. We represent multi-channel ECG signals as a high-dimensional tensor for anomaly detection. As opposed to existing approaches, we do not directly concatenate ECG signals into the image format or use ECG papers. Instead, a new time series imaging approach, i.e., Gramian Angular Field (GAF), is leveraged to cast multi-channel ECGs into a tensor, which preserves temporal and spectral correlation in the signal while maintain a high spatial interpretability.
2. A low-rank tensor decomposition approach is investigated to extract essential ECG features for unsupervised anomaly detection. Existing ECG analytics approaches are difficult to be used for extracting information from ECG tensor due to the high dimensionality and complex correlation structures. Additionally, most of them are supervised and are not suitable for IoHT-based monitoring. In this study, the multi-linear principal component analysis (MPCA) is investigated to unfold the ECG tensor and extract features to characterize disease-induced abnormal patterns. The developed tensor decomposition–unsupervised anomaly detection scheme provides great potential to realize personalized cardiac monitoring in the IoHT.
3. The developed framework is effective in detecting various disease-induced anomalies in the ECG. As opposed to many existing approaches that are designed for a specific type of cardiac disease, features unfolded from the ECG tensor contain rich information pertinent to a variety of disease-altered cardiac patterns. As such, the developed framework is effective in the identification of a broad range of cardiac disorders. This, in turn, makes it well-suited for IoHT-based monitoring.

The remainder of this paper is organized as follows: Section II presents details of the research methodology. Section III describes the datasets used in this study and the experimental design. Experimental results are shown in Section IV, and Section V includes the discussion and conclusions arising out of this study.

## 2. Research Methodology

**Figure 1:**
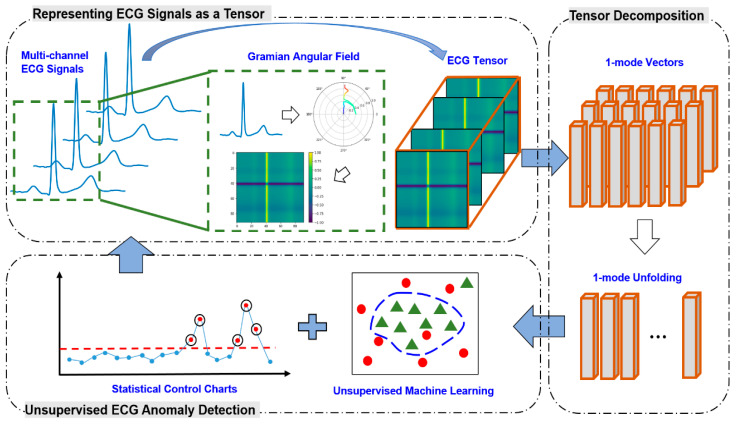
Overview of the proposed framework for tensor-based ECG anomaly detection.

The overview of research methodology is schematized in Figure 1. First, multi-channel ECG signals are converted into 2D images, which are further formulated as a high-dimensional tensor. Second, the ECG tensor is projected into low-dimensional representations, i.e., feature vectors, which capture a majority of variations in the original tensor. With the extracted feature vectors, an unsupervised machine learning model is leveraged for the detection of disease-induced abnormal patterns. Finally, a statistical control chart is constructed for real-time cycle-to-cycle monitoring of cardiac conditions.

### 2.1. Formulation of the ECG Tensor

Converting ECG signals to high-dimensional data provides a great opportunity to leverage a richer body of advanced machine learning methods for the identification of disease-altered cardiac patterns. For the converting process, the rule of thumb is to preserve, as much as possible, the temporal correlation in original ECG signals while enriching the spatial interpretability in the obtained images. As discussed above, most widely used existing approaches directly used ECG papers as images, which carry limited information from the original signals.

GAF is a recently developed approach that computes a Gramian matrix from the time series in the polar coordinate system [28]. The obtained matrix, in turn, can be considered as a 2D image for further processing. By denoting the ECG signal with length M as $x={\left(x_{1},x_{2},\;\ldots ,\;x_{M}\right)}^{T}$, it can be rescaled into the range of [0, 1] as:

(1) $${\tilde{x}}_{m}=\frac{x_{m}-\min\left(x\right)}{\max\left(x\right)-\min\left(x\right)}$$

The rescaled ECG can then be denoted as $\tilde{x}={\left({\tilde{x}}_{1},{\tilde{x}}_{2},\;\ldots ,\;{\tilde{x}}_{M}\right)}^{T}$. Further, the rescaled ECG is represented in the polar coordinate system. For the $m^{th}$ point ${\tilde{x}}_{m}$ in the signal, its angle $\varphi_{m}$ and radius $r_{m}$ are computed as:

(2) {φm=arccos(x˜m)rm=mM

As such, the ECG trace along the time is converted to the trajectory warping among the angles in polar coordinates. By exploring the angular variations, it provides a great opportunity to reveal underlying dynamics of the ECG signals. More importantly, it facilitates the generation of a Gramian matrix based on the angles. As discussed in [28], the Gramian Angular Difference Field (GADF), denoted as $G\in {\mathbb{R}}^{M\times M}$, can be defined as:

(3) $$G={\left[\sin\left(\varphi_{i}-\varphi_{j}\right)\right]}_{i,j}\;=\left[\begin{array}{ccc} \begin{array}{c} \sin\left(\varphi_{1}-\varphi_{1}\right)\\ \sin\left(\varphi_{2}-\varphi_{1}\right) \end{array} & \begin{array}{c} \ldots \\ \ldots \end{array} & \begin{array}{c} \sin\left(\varphi_{1}-\varphi_{M}\right)\\ \sin\left(\varphi_{2}-\varphi_{M}\right) \end{array}\\ \vdots & \ddots & \vdots \\ \sin\left(\varphi_{M}-\varphi_{1}\right) & \cdots & \sin\left(\varphi_{M}-\varphi_{M}\right) \end{array}\right]$$

Notably, if we define the inner product as $\langle u,\;v\rangle =\sqrt{1-u^{2}}\cdot v-u\cdot \sqrt{1-v^{2}}$, Equation (3) can be represented as [28,29]:

(4) $$G=\sqrt{1-{\tilde{x}}^{2}}\cdot {\tilde{x}}^{T}-\tilde{x}{\left(\sqrt{1-{\tilde{x}}^{2}}\right)}^{T}$$

where 1 is the unitary vector.

It may be noted that 𝒢 resembles a 2D image with ${\left[\sin\left(\varphi_{i}-\varphi_{j}\right)\right]}_{i,j}$as the value of the$\;{\left(i,j\right)}^{th}$ pixel. In other words, 𝒢 can be considered as a 2nd-order ECG tensor. Further, a stack of images from multiple ECG channels (e.g., 12 images from the 12-lead ECG signal) will form a 3rd-order ECG tensor, $A\in {\mathbb{R}}^{M\times M\times C}$, i.e., $A=G^{\left(1\right)}⊕G^{\left(2\right)},\;\ldots ,⊕G^{\left(C\right)}$, where $G^{\left(c\right)}$is the GADF image converted from the $c^{th}$ channel and C is the number of ECG channels. The ⊕ represents the operation of concatenation of the images along the 3rd dimension (see Figure 1).

**Figure 2:**
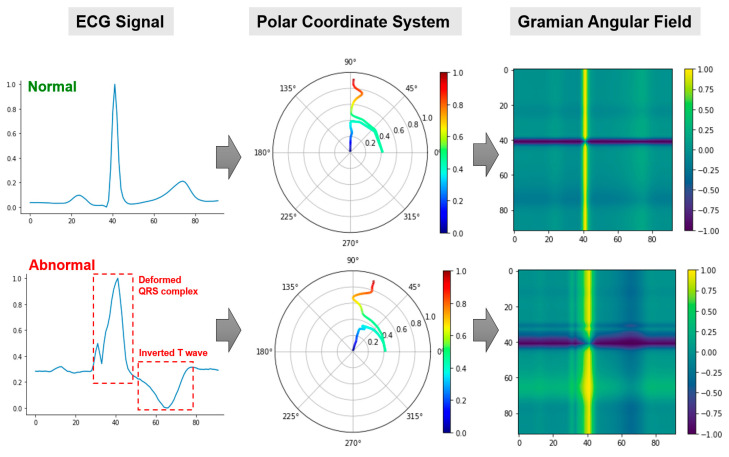
The construction of GADF images from 1D ECG signals. Left column: rescaled ECG signals; middle column: projected ECGs in the polar coordinate system; and right column: obtained 2D GADF images.

Figure 2 illustrates the process of transforming a single channel of ECG as a GADF image. Specifically, the rescaled ECG signal is first mapped into the polar coordinate system using Equation (2). Then, the GADF image is generated by calculating each element in the Gramian matrix in Equation (3). Notably, distinct traces are obtained when projecting the normal and abnormal ECG signals onto the polar coordinate system. The resulted GADF images are able to depict the variations between normal and abnormal ECG patterns. For example, the abnormal ECG is associated with a wider QRS complex with a small peak. This is reflected as a wider and intermittent yellow band in the GADF image. Additionally, the inverted T wave manifests as a dark band on the GADF image, as opposed to a light band for normal ECG.

Notably, a long ECG signal (i.e., a large M) may pose challenges on the construction and processing of the GADF image. Similar to [28], we adopt the piecewise aggregate approximation (PAA) strategy to reduce the ECG length while preserving its main morphology. The basic idea of the PAA is to divide the original time series (with length M) into $M^{\prime }$ equi-sized segments ($M^{\prime }\leq M$). By averaging the values in each segment, a new time series with a reduced length $M^{\prime }$ can be obtained. For more information about the PAA, please refer to [30].

### 2.2. Low-Rank ECG Tensor Decomposition

The ECG tensor contains important information pertinent to the underlying disease-altered cardiac electrical activity. Accurate detection of cardiac disorders hinges, to a great extent, on the information processing and feature extraction from the ECG tensor. Notably, off-the-shelf deep learning architectures, such as CNN, can be directly applied on the ECG images for disease pattern recognition. However, most deep learning architectures only focus on supervised learning, and they can hardly be interpreted due to the black-box nature (e.g., it is difficult to comprehend how the variables are combined to make the prediction due to the multilayer nonlinear structure of the model). To facilitate both supervised and unsupervised anomaly detection, a low-rank tensor decomposition approach, i.e., MPCA [31] is explored in this study. The objective of low-rank tensor decomposition is to obtain a compact representation of the high-dimensional tensor while preserving the correlation structure in the data [32]. The resulted low-dimensional representation, i.e., projected tensors, can be considered as features for both supervised and unsupervised anomaly detection.

Originating from the PCA, MPCA performs multilinear dimension reduction on tensor data. According to multilinear algebra, a $K^{th}$-order tensor, 𝒳 $\in {\mathbb{R}}^{I_{1}\times I_{2}\times \ldots \times I_{K}}$, can be represented as the following product:

(5) $$X=V\times_{1}U^{\left(1\right)}\times_{2}U^{\left(2\right)}\times \ldots \times_{K}U^{\left(K\right)}$$

where V is the low-dimensional projected tensor (i.e., the core tensor) and $U^{\left(k\right)}\in {\mathbb{R}}^{I_{k}\times P_{k}}$, $P_{k}<I_{k}$ is an orthogonal projection matrix. The objective of MPCA is to optimally determine a set of orthogonal projection matrices, $U=\left\{U^{\left(k\right)},\;k=1,\;2,\;\ldots ,K\right\}$, so that the projected tensor, V, captures the majority of variations in X [32]. For the present study, in particular, MPCA aims to maximize the total tensor scatter in the projected tensor $S^{\left(i\right)}$ as:

(6) $$\left\{U^{\left(1\right)},U^{\left(2\right)},U^{\left(3\right)}\right\}=\arg\underset{U^{\left(1\right)},\;U^{\left(2\right)},U^{\left(3\right)}}{\max}\overset{N}{\underset{i=1}{\sum }}\|S^{\left(i\right)}{\|}_{F}^{2}$$

and

(7) $$S^{\left(i\right)}=A^{\left(i\right)}\times_{1}U^{\left(1\right)}{}^{T}\times_{2}U^{\left(2\right)}{}^{T}\times_{3}U^{\left(3\right)}{}^{T}$$

In Equation (6), the total tensor scatter, $\|S^{\left(i\right)}{\|}_{F}^{2}$, is defined with the Frobenius norm, which is calculated based on the inner product of two tensors [32]. With the projected tensor, $S^{\left(i\right)}\in {\mathbb{R}}^{P_{1}\times P_{2}\times P_{3}}$, the $i^{th}$ ECG sample can finally be represented by a 1D feature vector, i.e., $\vartheta^{\left(i\right)}=\mathrm{vec}\left(S^{\left(i\right)}\right)$, where vec (·) denotes the vectorization operation. An iterative algorithm can be used to solve Equation (6) and obtain the orthogonal projection matrices, $U^{\left(1\right)},U^{\left(2\right)},U^{\left(3\right)}$. For more information about the optimization procedure, please refer to [31,33]. When a new ECG sample is collected, the tensor representation, $A^{\left(\mathrm{new}\right)}$, is firstly obtained using GADF. Then, the projected tensor can be calculated using Equation (7), $S^{\left(\mathrm{new}\right)}=A^{\left(\mathrm{new}\right)}\times_{1}U^{\left(1\right)}{}^{T}\times_{2}U^{\left(2\right)}{}^{T}\times_{3}U^{\left(3\right)}{}^{T}$. Finally, the feature vector of the new sample is obtained as $\vartheta^{\left(\mathrm{new}\right)}=\mathrm{vec}\left(S^{\left(\mathrm{new}\right)}\right)$.

### 2.3. Unsupervised ECG Anomaly Detection

To this end, it is imperative to quantitatively associate the feature vector, $\vartheta^{\left(i\right)}$, with the cardiac condition (normal vs. abnormal, or more specifically, the type of cardiac disease) for the purpose of anomaly detection. In this study, both unsupervised machine learning algorithm, i.e., deep support vector data description (deep SVDD) and statistical control chart, i.e., Hotelling $T^{2}$ are investigated for ECG anomaly detection. Developed by Tax and Duin [34], the objective of SVDD is to find the minimum-volume hypersphere that encloses the majority of the normal data in the feature space. Given a set of feature vectors from normal ECG tensors as $\vartheta^{\left(i\right)},\;i=1,2,\;\ldots ,\;N_{0}$, the primal problem of SVDD can be formulated as:

(8) $$\underset{R,\;c,\gamma \;}{\min}\left(R^{2}+\gamma \overset{N_{0}}{\underset{i=1}{\sum }}\xi^{\left(i\right)}\right)\;\mathrm{Subject}\;\mathrm{to}\;\|\phi \left(\vartheta^{\left(i\right)}\right)-{c\|}^{2}\leq R^{2}+\xi^{\left(i\right)},\;\xi^{\left(i\right)}\geq 0,\;\forall i=1,\;2,\;\ldots ,\;N_{0}$$

where c is the center and R is the radius of the hypersphere. The slack variables, $\xi^{\left(i\right)}$, allow a soft boundary, i.e., normal samples can be placed out of the hypersphere with penalty. The parameter γ controls the tradeoff between the volume of the hypersphere and the number of errors (i.e., the number of normal samples out of the hypersphere);$\;\phi \left(\vartheta^{\left(i\right)}\right)$ denotes a nonlinear mapping function. Solving Equation (8) requires the formulation of its dual problem, which incorporates a kernel function, $K\left(\vartheta^{\left(i\right)},\;\vartheta^{\left(j\right)}\right)$. In most cases, the Gaussian kernel is used, i.e., $K\left(\vartheta^{\left(i\right)},\;\vartheta^{\left(j\right)}\right)=\phi \left(\vartheta^{\left(i\right)}\right),\phi \left(\vartheta^{\left(j\right)}\right)=\exp\left(-\|\vartheta^{\left(i\right)}-\vartheta^{\left(j\right)}{\|}^{2}/2\sigma^{2}\right)$ [35].

Instead of directly learning the hypersphere, Ruff et al. [36] further trained a deep neural network to map the input into a latent space, in which the mappings of normal samples are located within, whereas mappings of anomalies are placed outside the hypersphere. This leads to the deep SVDD algorithm, which has been shown to outperform classic SVDD in anomaly detection [36]. The objective function of deep SVDD is formulated as:

(9) $$\underset{W}{\min}\frac{1}{N_{0}}\overset{N_{0}}{\underset{i=1}{\sum }}\|\phi \left(\vartheta^{\left(i\right)};W\right)-{c\|}^{2}+\frac{\lambda }{2}\;\overset{L}{\underset{l=1}{\sum }}\|W^{l}\|{}_{F}^{2}$$

where $\phi \left(\vartheta^{\left(i\right)};W\right)$ denotes a neural network model with L hidden layers, and $W=\left\{W^{1},W^{2},\ldots ,W^{L}\right\}$ is a set of weights, with $W^{l}$ denoting the weights of layer l. The first term minimizes the mean distance of all training (i.e., normal) samples to the center. The second term is a weight decay regularizer on the weights of the neural network, W. Here, λ controls the regularization and ${\left\|\cdot \right\|}_{F}$ represents the Frobenius norm. When a new sample is presented, an anomaly score can be obtained by measuring its mapping with respect to the center, i.e., $\|\phi \left(\vartheta^{\left(\mathrm{new}\right)},W^{*}\right)-{c\|}^{2}$, where $W^{*}$ denotes the trained network weights, which are obtained using stochastic gradient decent. For more details of deep SVDD, please refer to [36].

In addition to the unsupervised machine learning algorithm, statistical control charts can be leveraged for the unsupervised detection of disease-induced cardiac activity. Notably, a variety of control charting schemes can be used, and in this study we employ the Hotelling $T^{2}$ control chart on the obtained ECG feature vector (i.e., $\vartheta^{\left(i\right)}$). First, the mean and covariance matrix of in-control samples, i.e., ${\bar{\vartheta }}_{0}$ and $S_{0}$, are computed from the in-control feature set, $\vartheta^{\left(i\right)},\;i=1,2,\;\ldots ,\;N_{0}$. Then, given a new ECG signal, the feature vector, $\vartheta^{\left(\mathrm{new}\right)}$, will be obtained, as discussed in Section 2.2, and the Hotelling $T^{2}$ monitoring statistic can be calculated as:

(10) $$T^{2}={\left(\vartheta^{\left(\mathrm{new}\right)}-{\bar{\vartheta }}_{0}\right)}^{T}S_{0}^{-1}\left(\vartheta^{\left(\mathrm{new}\right)}-{\bar{\vartheta }}_{0}\right)$$

The upper control limit (UCL) of the $T^{2}$ control chart can be estimated using the empirical distribution of the $T^{2}$ statistics from the in-control samples, i.e., the $\left(1-\alpha \right)100^{\mathrm{th}}$ percentile obtained from the empirical distribution is used as the UCL [32].

## 3. Materials and Experimental Design

The proposed framework is evaluated and validated using real-world ECG data from: (1) the 2018 China Physiological Signal Challenge (CPSC2018) dataset [37] and (2) the PhysioNet Long-term ST dataset [38,39]. The CPSC2018 dataset contains 12-lead ECG recordings from 6877 patients. Each recording lasts from six seconds to one minute and is digitalized at 500 Hz sampling rate. A label, i.e., the diagnosis of cardiac condition, is assigned to each ECG recording by expert annotators. In total, nine conditions are included in the CPSC2018 dataset, and the number of recordings under each condition is provided in Table 1. The long-term ST dataset includes 86 ECG recordings that are 21 to 24 h in duration and exhibit ST-segment changes, such as slow ST level drift and ischemic ST episodes. Each ECG recording is digitalized at 250 Hz and contains two or three leads. Notably, each ECG cycle is reviewed by experts and assigned with a label (i.e., normal or a specific type of cardiac condition) in the long-term ST dataset.

In this study, we use the CPSC2018 dataset to evaluate the proposed framework with unsupervised machine learning approach, i.e., deep SVDD. Similar to [1], an ensemble cycle is obtained from each ECG recording by averaging all the cycles in that recording. The ensemble cycle is fed into the proposed framework, which determines if it is associated with normal or abnormal cardiac condition. For the long-term ST dataset, it is well-suited for implementing patient-centered cardiac monitoring because continuous ECG recordings for a few hours are included, and each ECG cycle has been assigned with a label. Therefore, the long-term ST dataset is used to evaluate the proposed framework with the Hotelling $T^{2}$ control chart on individual patients.

With the CPSC2018 dataset, we further conduct a sensitivity analysis to evaluate the performance of the proposed framework with respect to the size of the GADF image. It may be noted that the larger the GADF image, the more information can be captured from the original ECG signal. However, large GADF images will pose challenges in the low-rank tensor decomposition. It is important to investigate if the proposed framework is sensitive to the change in the size of GADF image. Moreover, the proposed framework is compared with a few benchmark approaches for performance evaluation. Specifically, three categories of benchmark approaches are considered:

1. Directly use 12-lead ECG without converting each lead into a GADF image. Notably, handcrafted ECG features have been extensively investigated for pattern recognition of cardiac disorders. Following [40], multiple time- and frequency-domain features are extracted from each ECG sample, which are then fed into the deep SVDD. We denote this benchmark approach as *12ECG + HandFeatures + D-SVDD*. Furthermore, existing studies have applied MPCA on multi-channel signals for feature extraction [41]. Again, the output of MPCA is processed by the deep SVDD. We denote this benchmark approach as *12ECG + MPCA + D-SVDD*. Furthermore, each 12-lead ECG sample is a 2D matrix, which can be directly processed by the deep SVDD. We denote this approach as *12ECG + D-SVDD*. The proposed framework is denoted as *GADF + MPCA + D-SVDD*.
2. Unsupervised learning without using the deep neural network. That is, after formulating ECG tensor and MPCA, the obtained low-dimensional projected tensor is fed into the classic SVDD algorithm in Equation (8). We denote this benchmark approach as *GADF + MPCA + C-SVDD*.
3. Supervised learning models. That is, instead of unsupervised learning, two widely used machine learning models for ECG analysis, i.e., Adaboost [42] and support vector machine (SVM) [43] are implemented. The models are trained with both normal and abnormal ECG samples with labels. We denote these benchmark approaches as *GADF + MPCA +* Adaboost and *GADF + MPCA + SVM*, respectively.

## 4. Results

Following the experimental design in Section 3, results for the sensitivity analysis and performance comparisons using the CPSC2018 dataset are firstly reported in Section 4.1. Further, Section 4.2 demonstrates the results of patient-centered cardiac monitoring using Hotelling $T^{2}$ control charts.

In this study, the performance of the proposed framework is evaluated by three widely-used metrics, i.e., accuracy, area under the ROC curve (AUROC) and the F-score. Given the true positive (TP), false positive (FP), true negative (TN), and false negative (FN) obtained from the anomaly detection result, the accuracy is calculated as:

(11) $$\mathrm{Accuracy}=\frac{\mathrm{TP}+\mathrm{TN}}{\mathrm{TP}+\mathrm{FP}+\mathrm{TN}+\mathrm{FN}}$$

The precision and recall, which are closely related to the Type I error and Type II error, respectively, are calculated as Precision = TP/(TP + FP) and Recall = TP/(TP + FN). With that, the F-score is obtained as:

(12) $$F-\mathrm{score}=2\cdot \frac{\mathrm{Precision}\cdot \mathrm{Recall}}{\mathrm{Precision}+\mathrm{Recall}}$$

In addition, the true positive rate (TPR) and false positive rate (FPR) can be computed as: TPR = TP/(TP + FN) and FPR = FP/(FP + TN). The receiver operating characteristic (ROC) curve plots TPR against FPR by varying classification thresholds, which can be summarized by the AUROC to show the ability of the model for predicting an abnormal sample.

### 4.1. Unsupervised Machine Learning for ECG Anomaly Detection

**Figure 3:**
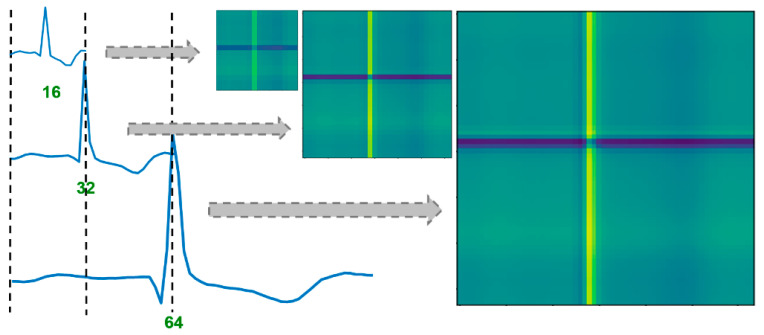
The effect of ECG length on the GADF image; the image generated from a longer ECG captures fine-grained patterns of the signal, and the image generated from a shorter ECG (length reduced using PAA) captures coarse-grained patterns.

We first evaluate the sensitivity of the developed framework with respect to the length of ECG (i.e., the size of the corresponding GADF image). Here, we vary the length of ECG (i.e., $M^{\prime }$) by tuning the PAA algorithm before generating the GADF image. As shown in Figure 3, fine-grained ECG patterns are well captured by the GADF image when the signal length is 64. The smaller GADF image (e.g., the one generated from ECGs with a length of 32) shows similar patterns as the 64 × 64 image, which preserves most information of the ECG signal. When the ECG length is further reduced to 16, the generated image is blurred and less smooth. Some patterns shown in the larger GADF images are diminished in the small image. Notably, even with such a small GADF image, the coarse-grained patterns that are induced by cardiac disease (e.g., ST elevation/depression) can still be revealed.

**Figure 4:**
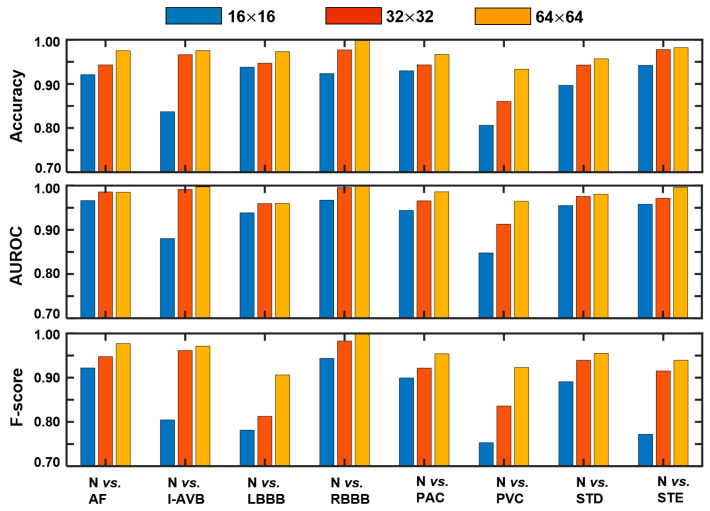
Performances of the proposed framework with varying size of the GADF image, i.e., 16 × 16, 32 × 32, and 64 × 64.

As shown in Figure 4, with larger GADF images, the proposed framework tends to achieve higher accuracy, AUROC, and F-score. Here, the first 10 principal components are kept in the MPCA (i.e., p= 10). It may be noted that over 90% in accuracy and AUROC are achieved by the proposed framework with the three image sizes under most of abnormal conditions. The three image sizes reach similar performances when a long segment of the ECG has been altered by the disease (e.g., the elevation of the ST segment). Nevertheless, it is difficult for a small GADF image to preserve fine-grained patterns in the detection of I-AVB and PVC.

Table 2 demonstrates the results of performance comparison with three benchmark approaches in Category 1. As shown in the results, it is not effective in detecting disease-induced abnormities by directly applying the deep SVDD on the 12-lead ECG matrix (i.e., 12ECG + D-SVDD). Furthermore, for hand-crafted features (i.e., 12ECG + HandFeatures + D-SVDD), they are effective in detecting some types of disease-altered patterns, e.g., AF and LBBB, whereas they are limited in their ability to identify I-AVB, PCA, and STD. This is because, in the literature, most hand-crafted ECG features are carefully designed according to a specific disease. In other words, some hand-crafted features are only effective for detecting certain types of cardiac disease. They are limited in the ability to identify other types of cardiac disorders. This, in turn, makes hand-crafted ECG features not well-suited for cardiac monitoring in the IoHT, because it is difficult to have the prior knowledge regarding what type of cardiac disease will occur before monitoring (see the discussion in Section 2). Moreover, applying MPCA directly on the 12-lead ECG signal (i.e., 12ECG + MPCA + D-SVDD) results in compromised performances for most types of cardiac disease. Finally, the developed framework achieves superior results for the detection of all types of cardiac diseases in the CPSC2018 dataset. To confirm, we have performed a paired *t*-test to evaluate if the mean of the F-scores achieved by the developed framework differs significantly from the F-scores achieved by each of the benchmark approaches (with a null hypothesis of equal means). A *p-*value of 0.0097, 0.0002, and 0.0022 is obtained from the paired *t*-test between the developed framework vs. 12ECG + HandFeatures + D-SVDD, 12ECG + MPCA + D-SVDD, and 12ECG + D-SVDD, respectively. Therefore, the performances of the developed framework are significantly better (under the level of significance α= 0.05) than the benchmark approaches in Category 1.

**Figure 5:**
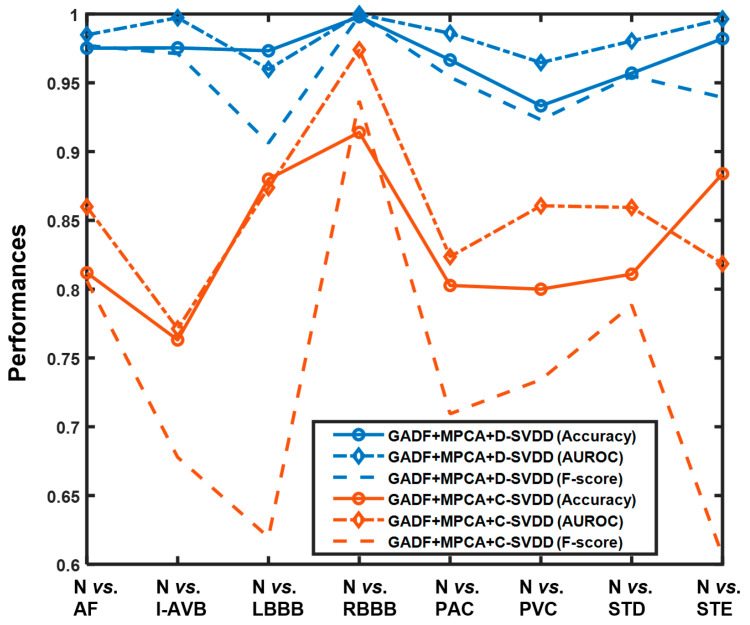
Performance comparison between the proposed framework, i.e., GADF + MPCA + D-SVDD and unsupervised learning without using the deep neural network, i.e., GADF + MPCA + C-SVDD in the benchmark, Category 2.

When comparing with conventional unsupervised learning approach (i.e., GADF + MPCA + C-SVDD in the benchmark, Category 2), the effectiveness of the developed framework is further highlighted. More importantly, the developed framework performs well in detecting all types of disease-induced cardiac anomalies in the CPSC2018 dataset. As shown in Figure 5, the performance of conventional SVDD fluctuates dramatically among different cardiac conditions, and most of the achieved F-scores are low (e.g., only around 0.61 and 0.6 F-scores are achieved for separating normal from LBBB and STE, respectively). On the contrary, the proposed framework that leverages deep SVDD achieves over 0.95 accuracy, AUROC, and F-score for most cases. The superb performance of deep SVDD is mainly due to the trained data-enclosing hypersphere [36].

Finally, the proposed framework is compared with supervised learning approaches (in the benchmark, Category 3) for anomaly detection. Notably, we still follow the proposed ECG tensor representation and MPCA for feature extraction and only change the deep SVDD to supervised models, i.e., Adaboost and SVM. Here, the radial basis function is used as the kernel function of the SVM. The regularization parameter is fixed as 1 and the gamma parameter is calculated as 1/(number of features × the variance of feature set), where the number of features (i.e., principal components) is p= 10. For Adaboost, each tree is split according to the Gini index, and the maximum depth is set as 14. As shown in Table 3, the proposed unsupervised anomaly detection framework and the supervised approaches achieve close performances for most disease types in the CPSC2018 dataset. To confirm, a paired *t*-test is performed to check if the mean of the F-scores obtained by the developed framework differs from that achieved by each of the benchmark approaches (with a null hypothesis of equal means). As a result, a *p*-value of 0.1366 and 0.4904 is obtained from the paired *t*-test between the developed framework vs. GADF + MPCA + Adaboost and GADF + MPCA + SVM, respectively. Therefore, close performances (without significantly different means under the level of significance α=0.05) are achieved for the developed framework as well as the two supervised learning approaches. This indicates that the proposed framework is effective in delineating disease-altered ECG patterns and separating them from normal patterns.

### 4.2. Patient-Centered Cardiac Monitoring

We leverage the PhysioNet long-term ST dataset to evaluate the performance of the developed framework for patient-centered cycle-based monitoring. Notably, IoHT-based heart monitors oftentimes collect ECG with fewer channels, e.g., 1-lead or 3-lead ECGs, to improve the efficiency in monitoring and data transmission. Thus, we only use one ECG channel to evaluate our framework. For a patient, the GADF + MPCA + Hotelling $T^{2}$ is implemented, in which $T^{2}$ statistics are calculated from successive ECG cycles for monitoring. Here, normal cycles from the first 5000 cycles (approximately 80 min) are considered as Phase-I data to calculate $U^{\left(1\right)},U^{\left(2\right)},U^{\left(3\right)}$ in Equation (6) and ${\bar{\vartheta }}_{0}$ and $S_{0}^{-1}$ in Equation (10), and construct the UCL of the control chart. This is practical as the first few hours of IoHT-based monitoring can be conducted under the supervision of cardiologists, and the patient-specific normal cycles can be manually identified. For illustration purpose, we only demonstrate the results of Phase II monitoring with 500 successive cycles of two patients in the dataset.

**Figure 6:**
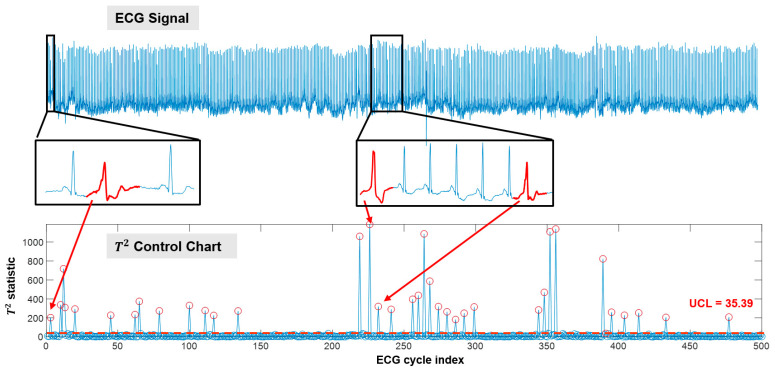
Cycle-based monitoring and anomaly detection for patient s20041 in the PhysioNet long-term ST dataset (only 500 cycles are demonstrated).

As shown in Figure 6, the developed framework is effective in detecting disease-altered ECG cycles when used in continuous monitoring settings. The upper panel of Figure 6 shows the ECG signal collected in approximately 8 min (500 cycles). The lower panel shows the corresponding Hotelling $T^{2}$ control chart. Based on the normal cycles in the first 80 min, the UCL is obtained as 35.39. During monitoring, when a new ECG cycle is collected, a $T^{2}$ statistic is calculated and plotted on the control chart. For the 500 cycles shown in Figure 6, the F-score achieved by the developed framework is 0.9863. It may be noted that the detected out-of-control cycles (marked as red circles on the control chart) are with abnormal morphological patterns. As shown in Figure 6, we zoom in on two segments of the ECG signal, and the detected abnormal cycles are highlighted in red. All three cycles demonstrate disease-altered patterns in the ST segment, which coincide with the labels provided by expert annotators. More importantly, cycles that are similar in morphology will obtain close $T^{2}$ statistics (see highlighted abnormal cycles in Figure 6), which indicates the effectiveness of the developed framework in delineating the morphological patterns of the ECG signal.

**Figure 7:**
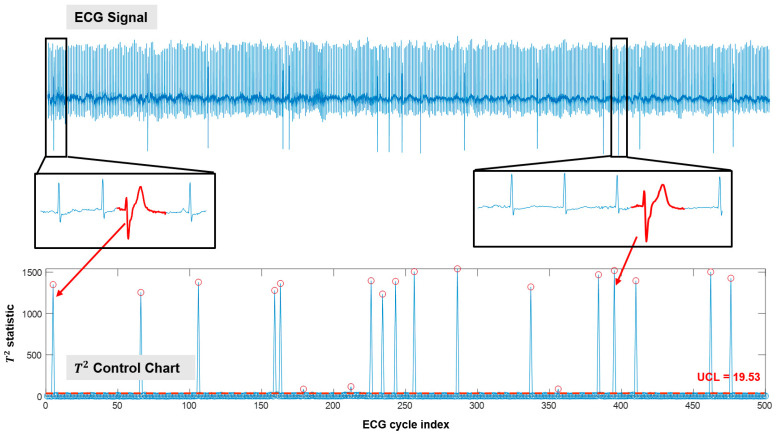
Cycle-based monitoring and anomaly detection for patient s20081 in the PhysioNet long-term ST dataset (only 500 cycles are demonstrated).

Figure 7 illustrates the cycle-based monitoring results for patient s20081 in the PhysioNet long-term ST dataset. Similar to Figure 6, ECG cycles with abnormal morphologies are detected as out-of-control cycles. Here, the UCL is obtained as 19.53 according to the Phase-I data (i.e., normal cycles in the first 80 min of monitoring) of this patient. The F-score achieved is 0.9143. Notably, if 19.53 is used as the UCL for patient s20041 (shown in Figure 6), more false alarms will be generated. This is because patient-to-patient variations exist even in the normal data. Thus, it is imperative to delineate the “normal condition” based on a patient’s own normal data for cardiac monitoring.

## 5. Conclusions and Discussion

In this study, a new framework is developed for unsupervised ECG anomaly detection. We first represent the multi-channel ECG as a 3rd-order tensor using GADF. Then, a low-rank tensor decomposition approach, i.e., MPCA, is leveraged to unfold the ECG tensor and extract essential features. Finally, an unsupervised machine learning approach, i.e., deep SVDD and statistical control chart, i.e., Hotelling $T^{2}$ chart, are implemented for cardiac condition monitoring and anomaly detection. The developed framework is extensively evaluated and validated on two real-world ECG datasets, i.e., CPSC2018 and PhysioNet Long-term ST. Experimental results have demonstrated the effectiveness of the proposed framework in detecting a variety of abnormal cardiac conditions, including AF, I-AVB, LBBB, RBBB, PAC, PVC, STD, and STE. Notably, the proposed framework works in an unsupervised manner, which has a great potential to be implemented in IoHT settings for personalized cardiac monitoring. This, in turn, will benefit the large population of cardiac patients by facilitating early identification of disease patterns and timely delivery of life-saving therapies.

Performances of the developed framework have been compared with three groups of benchmark approaches. First, the developed framework achieves better performances than those directly using 12-lead ECG signals. This is because tensor representation of ECG facilitates the delineation of temporal patterns of ECG trace in a high-dimensional space, which helps reveal hidden information that is difficult to extract directly from the 1D ECG. The MPCA approach is able to fully utilize the spatial and spectral information in the ECG tensor to highlight cycle-to-cycle variations from all ECG channels. As such, pertinent features about the underlying cardiac dynamics can be effectively extracted. On the contrary, even MPCA and deep SVDD can be directly applied on the 1D ECGs (e.g., the benchmark approach 12ECG + MPCA + D-SVDD), they are hampered in extracting sufficient information for the detection of abnormal ECG patterns. Second, the developed framework outperforms conventional unsupervised anomaly detection approaches (e.g., the benchmark approach GADF + MPCA + C-SVDD). This is mainly because the developed framework leverages the deep neural network to better cluster normal ECGs into the data-enclosing hypersphere. Third, the developed framework achieves comparable, if not better, results comparing to the supervised machine learning approaches, i.e., SVM and Adaboost. Usually, unsupervised learning approaches are less powerful in classification tasks than supervised learning approaches, because the latter utilize label information for training. Thus, the results obtained from the comparison further demonstrate the strong power of the developed framework in differentiating normal and abnormal ECG patterns. Although the developed framework incorporates multiple complex models, the computational burden is low. Once the framework is established, it can predict if the new ECG cycle is normal or abnormal within half a second, which meets the requirement of real-time monitoring as the heart rate is usually between 60 to 100 beats per minute. For detailed discussion on the computational complexity of GADF, MPCA, and deep SVDD, please refer to [28,31,36].

This study is among the first attempts to leverage GAF for tensorizing multi-channel ECG signals and further unfold the ECG tensor by low-rank tensor decomposition for unsupervised cardiac monitoring and anomaly detection. Notably, some previous studies have used GAF images for ECG analysis. For example in [44], the authors calculated the Hjorth parameters from ECGs, which were further mapped into 2D images using GAF. Nevertheless, the approach in [44] follows the ad-hoc “feature extraction and classification” scheme, with simple statistics (e.g., mean and skewness of the GAF image pixels) as features and K-nearest neighbors as the classifier. Such an approach is limited in its ability to fully exploit the hidden information for the identification of disease-altered ECG patterns, as complex cardiac dynamics are oftentimes present during IoHT-based cardiac monitoring. Additionally, our framework directly transforms each channel of the ECG signal into a GAF image. As such, high interpretability is preserved, as patterns of the GAF image are closely associated with the temporal variations in the ECG signal (see Figure 2). This is particularly important when implementing the developed framework in clinical practice.

There are some limitations of this study that need to be further explored in future work. For example, the ECG data used to evaluate the developed framework are not contaminated by noises. We need to improve the framework to enhance its robustness against instrumental and other type of noises. Additionally, although the developed framework shows a great potential to identify early signs of cardiac disorders, it has not been evaluated when the abnormal conditions considered in this study are fully developed. In addition, we will investigate if the proposed framework is effective when the abnormal ECG patterns are induced by multiple types of cardiac disorders jointly. This will pave the way for implementing a developed framework for monitoring high-risk patients after heart surgery.

## Figures and Tables

**Table 1:** ECG Data Included in the CPSC2018 dataset.

	Cardiac Condition	Count
1	Normal (N)	918
2	Atrial fibrillation (AF)	1098
3	First-degree atrioventricular block (I-AVB)	704
4	Left bundle branch block (LBBB)	207
5	Right bundle branch block (RBBB)	1695
6	Premature atrial contraction (PAC)	556
7	Premature ventricular contraction (PVC)	672
8	ST-segment depression (STD)	825
9	ST-segment elevation (STE)	202

**Table 2:** Performance Comparison with Benchmark Approaches in Category 1.

		N vs. AF	N vs. I-AVB	N vs. LBBB	N vs. RBBB	N vs. PAC	N vs. PVC	N vs. STD	N vs. STE
12ECG + HandFeatures + D-SVDD	Accuracy	0.9505	0.7600	0.9644	0.8623	0.7525	0.8254	0.5874	0.9018
	AUROC	0.9726	0.8261	0.9696	0.9031	0.8061	0.8671	0.5806	0.8602
	F-score	0.9552	0.7000	0.8889	0.8968	0.6726	0.7660	0.4586	0.5217
12ECG + MPCA + D-SVDD	Accuracy	0.6213	0.6492	0.8844	0.8375	0.6689	0.6825	0.6017	0.8616
	AUROC	0.6325	0.6630	0.7099	0.8897	0.6183	0.7042	0.6021	0.6313
	F-score	0.7119	0.5440	0.4800	0.8850	0.3265	0.6063	0.6293	0.4364
12ECG + D-SVDD	Accuracy	0.7129	0.6985	0.9467	0.8681	0.6823	0.7143	0.5387	0.9018
	AUROC	0.7555	0.7153	0.9183	0.9190	0.6287	0.7259	0.5005	0.8094
	F-score	0.7264	0.6288	0.8065	0.9021	0.3066	0.5982	0.3264	0.5417
GADF + MPCA + D-SVDD (proposed)	Accuracy	0.9752	0.9754	0.9733	0.9981	0.9666	0.9333	0.9570	0.9821
	AUROC	0.9849	0.9973	0.9598	0.9999	0.9860	0.9647	0.9804	0.9964
	F-score	0.9771	0.9712	0.9062	0.9986	0.9541	0.9231	0.9550	0.9394

**Table 3:** Performance Comparison with Supervised Learning Approaches in the Benchmark, Category 3.

		N vs. AF	N vs. I-AVB	N vs. LBBB	N vs. RBBB	N vs. PAC	N vs. PVC	N vs. STD	N vs. STE
GADF + MPCA + Adaboost	Accuracy	0.9955	0.9994	0.9582	0.9983	0.9973	0.9978	0.9966	0.9987
	AUROC	0.9971	0.9995	0.9851	0.9994	0.9966	0.9973	0.9966	0.9969
	F-score	0.9959	0.9993	0.8440	0.9987	0.9964	0.9973	0.9965	0.9953
GADF + MPCA + SVM	Accuracy	0.9406	0.9323	0.9867	0.9618	0.9599	0.9651	0.9799	0.9509
	AUROC	0.9928	0.9972	0.9976	0.9944	0.9970	0.9934	0.9992	0.9850
	F-score	0.9442	0.9272	0.9552	0.9716	0.9478	0.9572	0.9791	0.8451
GADF + MPCA + D-SVDD (proposed)	Accuracy	0.9752	0.9754	0.9733	0.9981	0.9666	0.9333	0.9570	0.9821
	AUROC	0.9849	0.9973	0.9598	0.9999	0.9860	0.9647	0.9804	0.9964
	F-score	0.9771	0.9712	0.9062	0.9986	0.9541	0.9231	0.9550	0.9394

## Data Availability

Publicly available datasets were analyzed in this study. The data can be found at http://2018.icbeb.org/Challenge.html and https://archive.physionet.org/physiobank/database/ltstdb/.
